# Performance comparison of the cobas Liat and Cepheid GeneXpert systems for *Clostridium difficile* detection

**DOI:** 10.1371/journal.pone.0200498

**Published:** 2018-07-24

**Authors:** Paul A. Granato, Glen Hansen, Emily Herding, Sheena Chaudhuri, Shaowu Tang, Sachin K. Garg, Catherine R. Rowell, Joanna Jackson Sickler

**Affiliations:** 1 Department of Clinical Microbiology, Laboratory Alliance of Central New York, Syracuse, NY, United States of America; 2 Department of Microbiology and Molecular Diagnostics, Hennepin County Medical Center, Minneapolis, MN, United States of America; 3 Department of Microbiology and Molecular Biology, Hennepin County Medical Center, Minneapolis, MN, United States of America; 4 Medical and Scientific Affairs, Roche Molecular Diagnostics, Pleasanton, CA, United States of America; 5 Department of Laboratory Device Trials, Laboratory Alliance of Central New York, Syracuse, NY, United States of America; Institut Pasteur, FRANCE

## Abstract

*Clostridium difficile* infection (CDI) is a high burden and significant cause of healthcare-acquired infectious diarrhea in the United States (US). Timely and accurate diagnosis of CDI enables the rapid initiation of antibiotic therapy and infection control policies to minimize disease transmission. Polymerase chain reaction (PCR) assays have become a preferred modality for diagnosing CDI in the US. The cobas Liat Cdiff PCR test is a novel assay that can be performed on-demand for hospital-based testing with a rapid 20-minute turnaround time from specimen collection to result reporting. We compared the clinical performance of the cobas Liat Cdiff test to the previously introduced Xpert *C*. *difficile*/Epi test; both tests are FDA-cleared PCR assays that detect the toxin B (*tcd*B) gene of *C*. *difficile*. Prospectively collected and remnant stool specimens from 310 patients with suspected CDI were obtained for analysis. The cobas Liat Cdiff and Xpert PCR tests showed an overall percent agreement of 97.4% (302/310; 95% CI: 95.0–98.9). Low bacterial burdens of toxigenic *C*. *difficile*, indicated by significantly delayed PCR cycle threshold (Ct) values, explained most of the discordance. Positive and negative percent agreement of the cobas Liat Cdiff test compared to the Xpert PCR test were 94.5% (52/55) and 98.0% (250/255), respectively. The clinical performance of the cobas Liat Cdiff test, combined with its simplicity of use and rapid result reporting, provides a reliable option for clinical laboratory use.

## Introduction

*Clostridium difficile* infection (CDI) is among the most frequently reported nosocomial diseases [1], responsible for 15% to 20% of antibiotic-related cases of diarrhea [2] and a major cause of antibiotic-associated pseudomembranous colitis [3]. Over the past decade, increasing clinical recognition of CDI coupled with the emergence of hypervirulent strains and the use of diagnostics that are better able to detect toxin-producing *C*. *difficile* has led to changes in the understanding of CDI epidemiology. Currently, over half a million new infections and 29,000 deaths are attributed to CDI each year in the United States alone [4]. Thus, laboratory tests that can quickly and accurately detect the presence of toxin-producing *C*. *difficile* in symptomatic patients are essential in providing the appropriate clinical and laboratory response to suspected cases of CDI. Accurate diagnosis of CDI is increasingly viewed as essential in supporting early antibiotic management and the implementation of infection control procedures needed to minimize *C*. *difficile* transmission [5].

Nucleic acid amplification tests (NAATs), such as polymerase chain reaction (PCR) assays, have become the preferred modality for diagnosing CDI in the United States. This preference has been largely due to their speed as well as their high sensitivity and specificity when used alone or as part of a 2-step algorithm with toxin-detecting enzyme immunoassays [6,7]. Several NAAT PCR assays that detect the toxin B gene (*tcd*B) of *C*. *difficile* have been cleared by the FDA for diagnostic use [8]. One such test, the cobas Liat Cdiff nucleic acid test performed on the cobas Liat System (cobas Liat Cdiff; Roche Diagnostics, Pleasanton, CA), offers a rapid, 20-minute, specimen-to-answer, qualitative, real-time PCR result for detecting the *tcdB* gene [9].

The cobas Liat Cdiff test represents a novel, rapid-cycling, PCR assay that can be easily performed as an on-demand test by all laboratory shifts. However, experience in clinical practice has not yet been evaluated. In this study, we compared the performance of two FDA-cleared PCR assays, the cobas Liat Cdiff test and the Xpert *C*. *difficile*/Epi test (Xpert PCR; Cepheid, Sunnyvale, CA), for their ability to detect the *tcdB* gene in freshly collected stool specimens from patients with suspected CDI. The Xpert PCR test and the cobas Liat Cdiff assays provide laboratories and clinicians with reliable and rapid detection of toxin-producing *C*. *difficile*. We hypothesized that the cobas Liat assay will compare favorably to the Xpert PCR assay in clinical performance and provide a further improved option for rapid (20 minute) detection of toxin-producing *C*. *difficile*.

## Methods

Between August and November 2017, 310 prospectively collected, de-identified, remnant stool specimens were tested in this study from patients with signs and symptoms of CDI. The patients were from two geographically distinct regions of the United States where the testing was performed—Laboratory Alliance of Central New York (LACNY) in Syracuse, New York, and Hennepin County Medical Center (HCMC) in Minneapolis, Minnesota. Of the 310 specimens evaluated, 259 were tested by LACNY while the remaining 51 specimens were processed by HCMC. The stool specimens were tested on the day of collection at each laboratory using both the Xpert PCR and cobas Liat Cdiff tests. Since stool specimens were residual and de-identified, written informed consent was not obtained from patients. Study protocols were approved by each study site’s respective Institutional Review Boards (LACNY: Schulman IRB #201705250; HCMC: Human Subjects Research Committee #17–4412) prior to the start of the study.

Specimens were tested from patients suspected of having CDI based on each study site’s established acceptance criteria. Both study sites required patients to have a history of diarrhea and no less than three loose stool movements per day for at least two days; individuals aged < 1 year were excluded from enrollment. Commonly accepted laboratory criteria also require that specimens be able conform to the shape of the collection container [10]. Therefore, testing was performed only on unformed stool specimens with a minimum volume of 2 mL; stool specimens that were formed or semi-formed were excluded from the study protocol. Specimens from previously enrolled patients were excluded from the study. After testing with the cobas Liat Cdiff and Xpert PCR tests, the remainder of each specimen was then stored at 2°C to 8°C. Within 120 hours of testing, the remnant specimens with discordant results (between cobas Liat Cdiff and Xpert PCR) were sent to a reference laboratory (TriCore Laboratories, Albuquerque, NM) for arbitration testing using an alternative molecular method (BD Max Cdiff test; Becton Dickinson and Co, Franklin Lakes, NJ). Specimens received at the reference laboratory were stored at 2°C to 8°C prior to testing.

Based on available performance data, an estimated 300 total specimens with a minimum of 50 with positive results according to the reference method were required to determine statistically significant overall agreement between the cobas Liat Cdiff and Xpert PCR tests. Overall percent agreement (OPA), positive percent agreement (PPA), and negative percent agreement (NPA) between the cobas Liat Cdiff and Xpert PCR tests were calculated with 95% score binomial confidence intervals (CIs). Adjusted performance data for the cobas Liat Cdiff test were then calculated based on arbitration testing performed with the BD Max Cdiff test.

Cycle threshold (Ct) values, which have been shown to inversely correlate with *C*. *difficile* bacterial toxin burdens [11], were recorded for the cobas Liat Cdiff and Xpert PCR tests to perform additional discordant analyses. Ct values for specimens with positive PCR values on Xpert PCR and/or cobas Liat Cdiff were normalized (using mean cobas Liat Cdiff values for all specimens with a positive cobas Liat Cdiff result and mean Xpert PCR values with discordant Xpert PCR positives) and their distribution was plotted graphically; results were stratified by their concordance or discordance. All analyses were performed using SAS/STAT^®^ software (SAS, Cary, NC).

## Results

Table 1 compares the performance results between the cobas Liat Cdiff and Xpert PCR tests. Of the 310 specimens tested, 55 (17.7% of all specimens) were *C*. *difficile* positive according to the Xpert PCR test, of which 44 (80% of positives) were from LACNY while 11 (20% of positives) were from HCMC. OPA, PPA, and NPA between the cobas Liat Cdiff and Xpert PCR test were 97.4% (302/310; 95% CI, 95.0–98.9), 94.5% (52/55; 95% CI, 84.9–98.9), and 98.0% (250/255; 95% CI, 95.5–99.4), respectively. The BD Max Cdiff test results matched the cobas Liat Cdiff test results for 1 of 5 cobas Liat Cdiff discordant positive specimens and 2 of 3 cobas Liat Cdiff discordant negative specimens. All data used for this analysis can be found in S1 Dataset.

**Table 1:** Comparison of the cobas Liat Cdiff and Xpert PCR tests.

		Xpert PCR Test Results, n		Total
		Positive	Negative	
**cobas Liat Cdiff test results, n**	**Positive**	52	5	57
	**Negative**	3	250	253
	**Total**	55	255	310
PPA (95% CI), %		94.5 (84.9–98.9)		
NPA (95% CI), %		98.0 (95.5–99.4)		
OPA (95% CI), %		97.4 (95.0–98.9)		

CI, confidence interval; NPA, negative percent agreement; OPA, overall percent agreement; PPA, positive percent agreement.

**Fig 1:**
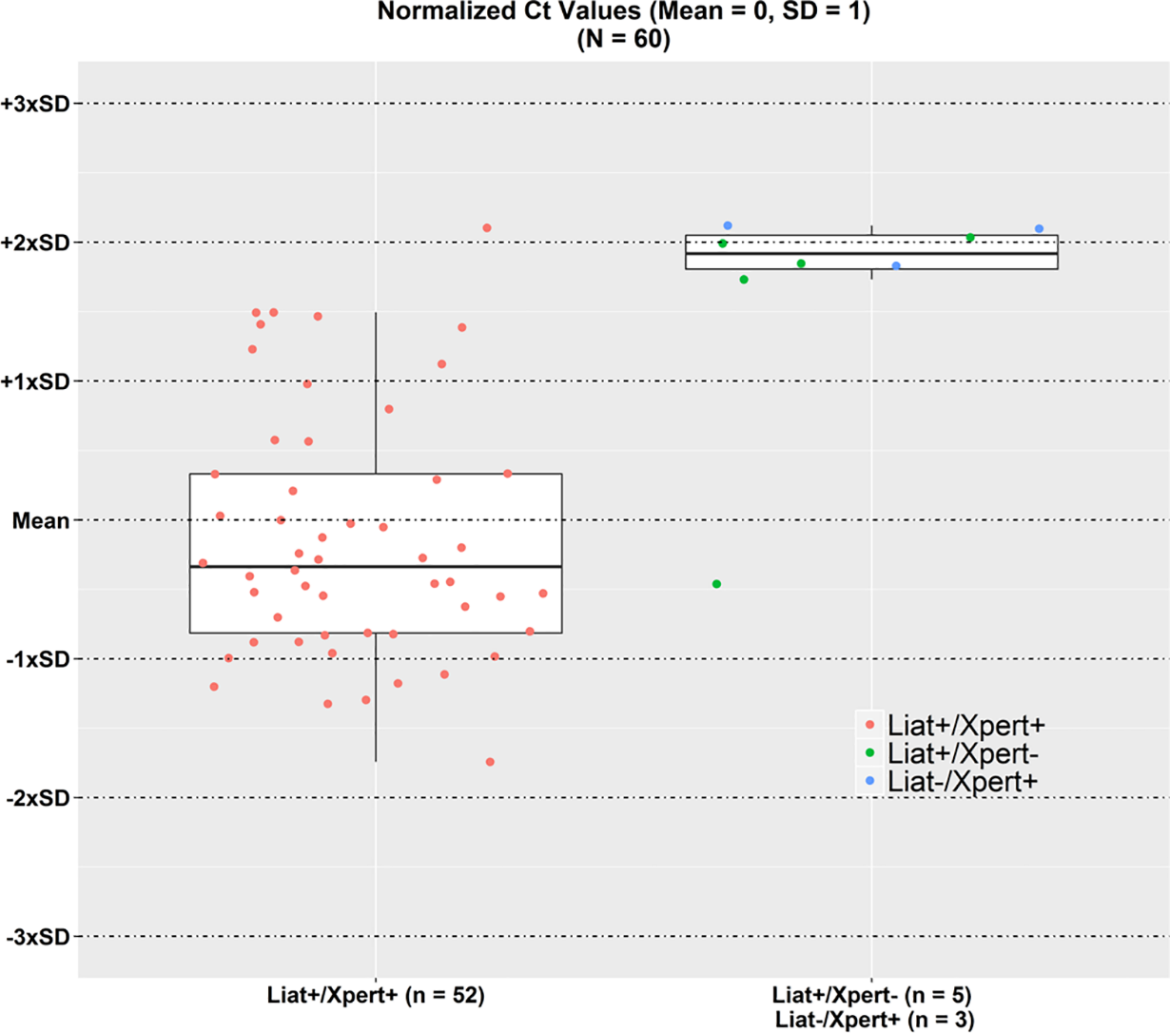
Normalized Ct values for point-of-care polymerase chain reaction *Clostridium difficile* concordant positive (n = 52) and discordant (n = 8) results. Ct, cycle threshold; Liat, cobas Liat Cdiff test; Xpert, Xpert PCR test.

Fig 1 shows the normalized Ct values for the positive results (n = 57) and for specimens with negative cobas Liat Cdiff results/positive Xpert PCR results (n = 3). Seven of 8 discordant samples had Ct values > 2 times the SD from the mean, indicating that low bacterial burden was likely the reason for the discordant results. These samples were close to the cutoff for the respective assay for a positive result. The eighth and remaining discordant specimen (cobas Liat Cdiff positive, Xpert PCR negative) had an earlier Ct value close to the mean cobas Liat Cdiff Ct value; testing with the BD Max Cdiff assay for this specimen also yielded a positive result.

## Discussion

We demonstrated high (> 97%) overall test concordance of the cobas Liat Cdiff and Xpert PCR tests for the detection of *C*. *difficile tcdB* gene in stool specimens. This is the first study to directly compare the clinical performance of both tests in routine clinical practice using patient stool specimens obtained from symptomatic patients. Several prior studies have compared the performance of other molecular assays for rapid CDI detection [8,12–16], two of which reported lower concordance rates (than those observed in our study) of 93% (75/81) [8] and 90% (306/339) [16] compared with the Xpert PCR test.

Discordance analysis further demonstrated a comparable performance of the cobas Liat Cdiff and Xpert PCR tests; 88% (7/8) of discordant specimens contained lower densities of toxigenic bacteria as indicated by significantly delayed (or higher) Ct values, which have been correlated with less severe CDI [17]. Arbitration of discordant test results with the BD Max Cdiff test produced mixed results. One partial explanation could be random variations in detection that occur as bacterial densities approach the assay limit of detection. Another explanation could be real differences in assay sensitivity. Previously performed studies using toxigenic culture as the reference method have independently established comparable sensitivities (> 90%) of the Xpert PCR [18–20] and cobas Liat Cdiff tests [9,21], but much lower sensitivity (< 90%) of the BD Max Cdiff test [22,23]. Despite this lower sensitivity, we chose to use the BD Max Cdiff test for arbitration because it is a readily available, widely used, and FDA-cleared NAAT for detection toxin B gene (*tcdB*) of *C*. *difficile*.

Comparison to toxigenic culture is a contemporary gold standard which was not assessed in our series. Toxigenic culture remains the most sensitive method for the isolation of *C*. *difficile* and subsequent confirmation of toxin-producing *C*. *difficile*. However, extended turnaround times, the need for quality control of specialized media, and requirements for staff labor make toxigenic culture less practical in today’s diagnostic laboratory environments. Practical laboratory data comparing various molecular platforms, such as the Xpert PCR and cobas Liat Cdiff tests in this study, is vital to the decision-making process for clinical laboratories.

The clinical performance of the cobas Liat Cdiff is equivalent to the Xpert PCR. Compared to Xpert PCR and other molecular assays, the cobas Liat Cdiff test provides an even shorter (20 minute) turnaround time and is a fully integrated, portable system that does not require an additional interface to perform testing [9]. The cobas Liat Cdiff test’s simplicity of use, rapid result reporting, and high sensitivity for *C*. *difficile tcdB* gene detection offer an additional sensitive and reliable technological option for small- and medium-sized laboratories to perform this diagnostic service. Additional studies are needed to further demonstrate how on-demand NAATs, such as the cobas Liat Cdiff test, can help reduce the time to diagnosis, improve CDI management, and contain the spread of infection both within and outside the healthcare setting.

## Supporting information

S1 Dataset Performance comparison of the cobas liat and cepheid GeneXpert systems for *Clostridium difficile* detection. (XLSX)
